# The Effect of Relevance on Children's Multiple Text Reading: Evidence From Eye Movements

**DOI:** 10.1111/sjop.70099

**Published:** 2026-04-16

**Authors:** Tuomo Häikiö, Oksana Kanerva, Norbert Erdmann, Mirjamaija Mikkilä‐Erdmann, Johanna K. Kaakinen

**Affiliations:** ^1^ Department of Psychology and Speech‐Language Pathology University of Turku Turku Finland; ^2^ Faculty of Arts, University of Helsinki University of Helsinki Helsinki Finland; ^3^ Department of Teacher Education University of Turku Turku Finland; ^4^ INVEST Research Flagship and Research Center University of Turku Turku Finland

**Keywords:** children, eye tracking, multiple text comprehension, reading, relevance

## Abstract

We examined how Finnish children read and integrate information across multiple expository texts when given an inquiry task. We were interested in how task‐relevance of text information affects readers' eye movements and whether the eye movements are connected to the quality of an essay written after reading. We were also interested in differentiating between the effects of technical reading skill and reading comprehension in respect to these processes. In total, 24 5th and 6th grade Finnish native‐speakers completed the experiment. Prior to testing, the participants were told that at the end of the testing session, they would have to complete an inquiry task (e.g., “What's the difference between human and dog hearing?”). During an eye tracking experiment, the participants read two science texts on the topic of the inquiry task. The texts contained both task‐relevant and task‐irrelevant text segments. After the reading task, the children wrote an essay to complete the inquiry task. Furthermore, participants' technical reading skill and reading comprehension were measured with an independent classroom test. It was shown that the task‐relevant segments were read longer than the task‐irrelevant segments during first‐pass reading. Moreover, reading skills modulated the effect of relevance, as weaker comprehenders were less likely to regress within an irrelevant segment. Furthermore, the relevance effect was more pronounced for the better technical readers with respect to look‐backs. No reliable effects were found for the essay‐writing task. The results imply that the participants were able to detect which parts of the text were relevant and adjusted their reading accordingly, based on their reading skills. However, they did not seem to form a coherent memory representation of the relevant text contents in order to perform well in the essay writing task.

## Summary

- Fifth and sixth grade Finnish children read science texts with an inquiry task in mind.
- Texts contained both task‐relevant and task‐irrelevant text segments.
- Eye movements revealed longer reading times for task‐relevant segments.
- Individual technical reading and comprehension skills modulated relevance effects.
- Children detected relevant parts, but this did not translate to better essay writing.

## 1 Introduction

While basic reading skills related to decoding are mostly set at an early age, comprehension and strategic reading skills keep developing until a later age. Strategic reading skills are essential when students seek and read information to acquire new knowledge, especially if information on the same topic is presented in more than one text. Hence, beyond single text comprehension, the reader also has to compare and integrate information across multiple texts which mutually reinforce, complement, or may even contradict each other (Magliano et al. 2018). In the present study, we examined how Finnish 5th and 6th grade children read and integrate information across two complementary expository texts when given an inquiry task. Finland, being a Nordic country where, according to the latest PISA results (OECD 2023), pupils have above average reading skills, offers an interesting testing ground for a reading task requiring higher order text processing. We focused on how task‐relevance of text information impacts readers' eye movements during reading, and whether the eye movements predict the quality of the written responses to the inquiry task. Furthermore, we were interested in disentangling the effects of technical reading skill and reading comprehension in these processes.

### 1.1 Multiple Document Comprehension

The MD‐TRACE model (Multiple‐Document Task‐based Relevance Assessment and Content Extraction; Britt et al. 2022; Rouet and Britt 2011; Rouet et al. 2017) considers factors that affect comprehension of multiple texts. According to MD‐TRACE, readers first develop a mental model of the task demands, a task model. This model includes a task goal, actions to achieve the goal (i.e., subgoals and procedures for achieving these goals), and a set of criteria for reaching those goals. For example, when given an inquiry task, such as “write a short essay on how dog and human hearing are similar or different,” the overall task goal is to be able to write a short essay on the given topic. This task may be divided into subgoals, such as searching for information about how dogs and humans hear, selecting task‐relevant texts to be read, processing information in each selected document by integrating text information with prior knowledge (Kintsch 1988; Kintsch and Van Dijk 1983), and, finally, integrating information thus constructed to complete the writing task. Readers then assess the task product. If it does not meet the criteria set in the task goal, they may return to previous phases of the process until the task demands are sufficiently met.

MD‐TRACE posits that assessing and determining relevance is crucial in successful text comprehension (Britt et al. 2022; Rouet and Britt 2011; see also Lee and List 2023). For example, one has to define relevant search terms when searching for information, select relevant documents for reading, identify and comprehend relevant information in the texts, integrate information across documents, and determine whether the task product contains enough task‐relevant information so that it fulfills the task demands as defined in the task model. The importance of relevance determinations has been documented in previous empirical studies with adults (e.g., Anmarkrud et al. 2013; Bråten et al. 2018; Lee and List 2023). For example, Anmarkrud et al. (2013) examined how university students read and comprehend information presented in multiple documents. Information judged to be relevant was more likely to be integrated with information presented in the other documents. Moreover, the relevance determinations were reflected in the quality of the essay written after the reading task. Bråten et al. (2018) showed that content relevance impacts document selection, reading time, and inclusion of information in an essay written after reading. The effects of content relevance were found regardless of topic familiarity, indicating that relevance determinations have a strong impact on multiple text comprehension.

### 1.2 Task Effects on Text Processing and Comprehension

The task model a reader has formed plays an important role in text comprehension (Lee and List 2023). First, the task model contains the *standards of relevance* (Lehman and Schraw 2002; McCrudden et al. 2011), referring to the criteria that needs to be met for the information to be considered relevant. For example, when given an inquiry task to compare human and dog hearing, standards of relevance probably define that information about humans and dogs is relevant, whereas information about fish or birds is not. Standards of relevance guide how processing resources are directed during reading: when readers recognize what information is relevant to their task, they spend more time reading task‐relevant than task‐irrelevant text segments (e.g., Rouet et al. 2001). Previous eye tracking studies suggest that adult readers are more likely to reread task‐relevant than task‐irrelevant sentences during first‐pass reading (i.e., time spent reading the sentence before it has been exited for the first time, with rereading referring to going back in the sentence during this time) and make more look‐backs to task‐relevant than task‐irrelevant sentences (e.g., Kaakinen et al. 2002; McCrudden et al. 2010). Selective processing of task‐relevant information during reading is also reflected in better recall of task‐relevant than task‐irrelevant text information in subsequent memory performance (e.g., Kaakinen et al. 2002; León et al. 2019; McCrudden et al. 2010).

Second, the task model includes the *standards of coherence* the reader maintains during reading (van den Broek 2010; van den Broek et al. 2002, 2011). They refer to the quality of the memory representation the reader aims to create, influencing the amount and nature of inferential activities during reading. For example, in response to an inquiry task about the similarities and differences between human and dog hearing, a reader sets the standards of coherence so that this comparison task can be successfully completed. If the available texts do not directly address the similarities and differences, the reader must integrate information from different sources with their prior knowledge to form an understanding of how human and dog hearing differ. The standards of coherence are set higher for task‐relevant information, reflected in reading behavior. Rouet et al. (2001) demonstrated that when adult readers encountered a task‐relevant text segment, they spent more time reading it than irrelevant segments. While this is similar to standards of relevance, if a reader has higher standards of coherence, it should also be reflected in their memory representation. In Rouet et al. (2001), the relevance effect was also dependent on the type of question; when questions did not demand integration of several text segments, only segments directly associated with the question were considered. When questions demanded integration across text segments, readers read more broad sections containing information relevant for the question.

In summary, the task the reader has in mind sets the standards for relevance and coherence, which are reflected in selective processing of task‐relevant information and better memory representation for the task‐relevant information. While most of the previous research has focused on adult readers, much less is known about task effects in children's reading.

### 1.3 Influence of Reading Skill on Task Effects

Previous studies suggest that also children are sensitive to task‐relevance during reading (e.g., Ballenghein and Lachaud 2025; Hautala et al. 2019), but the effects might depend on reading skill (e.g., van den Broek et al. 2009; van der Schoot et al. 2008). Studies that have examined whether children adjust their reading behavior to the reading task indicate that already 4th graders are sensitive to task relevance (Ballenghein and Lachaud 2025; Hautala et al. 2019; Schumacher et al. 1983). In their seminal study, Schumacher et al. (1983) examined how questions influence the eye movement patterns of 5th grade children and adults. For children, questions increased looking back in text. However, look‐backs were not particularly directed to question‐relevant text segments. On the other hand, for adults, look‐backs were directed to relevant segments. The authors concluded that children were aware of the task as their look‐backs were initiated from the questions but simply could not locate the relevant segments. In contrast, Hautala et al. (2019) suggested that 6th graders can locate task‐relevant information and tend to reread and look back to it in text. In their study, children made more and longer first‐pass reinspections (i.e., going back in the sentence before it has been exited for the first time) as well as look‐backs to task‐relevant sentences. In a recent study by Ballenghein and Lachaud (2025), French 4th and 5th grade children read one relevant and one irrelevant text while their eye movements were recorded. The relevance was based on which of the two topics (one text concerned water cycle and the other day/night cycle) the participants were told to learn as much as possible. It was shown that there were more fixations and longer total reading time on the relevant texts than the irrelevant ones. Furthermore, the participants scored better in reading comprehension tests for relevant than irrelevant texts.

However, previous research shows that reading skill is important in how efficiently readers can attend to task‐relevant information in text (e.g., van den Broek et al. 2009; van der Schoot et al. 2008). In van der Schoot et al. (2008), 5th and 6th grade children read texts while they had to take a certain perspective (science journalist vs. gossip journalist). The texts contained science words (e.g., plankton) and gossip words (e.g., affair), with science words being relevant for the science journalist perspective and irrelevant for the gossip journalist perspective, and vice versa. Relevant words elicited longer fixation durations both for first‐pass reading and look‐backs (called regressive fixation duration in their paper). Furthermore, better comprehenders made relatively longer look‐backs into relevant vs. irrelevant words. The authors concluded that more skilled comprehenders were more aware of the importance of reading relevant words again than less skilled comprehenders.

Looking at reading patterns across text segments, van den Broek et al. (2009) showed that reading skill is important in selective look‐backs to task‐relevant text segments. In their study, 4th, 7th, and 9th grade children read long texts. While the number of fixations was the same for struggling and proficient readers of the same age, struggling readers made longer fixations. While reading skill did not modulate the number of look‐backs, skilled readers made look‐backs especially to relevant segments while less skilled readers were not systematic in directing their look‐backs.

In a recent study by Chen et al. (2025), Mandarin Chinese speaking 4th grade children read sets of two texts presented simultaneously on the computer screen. In one block, their task was to read for general comprehension and in another block to integrate the contents of the two texts. For assessing integrative processes, entropy was calculated to measure transitions between the two texts. In this context, higher entropy refers to a higher number of shifts between different parts of texts reflecting more integration attempts than lower entropy. It was shown that in the integration condition, the entropy was higher than in the general comprehension condition for first pass reading and overall. Moreover, those children with higher reading comprehension had a higher entropy during second pass reading in the integration condition than in the general comprehension condition. There was no such effect for those with lower reading comprehension. Chen et al. concluded that the pattern of results indicated that while all children were affected by the reading task, children with better reading comprehension were better at using more purposeful integration strategies when the task explicitly required integration. Finally, using the same set of texts, Chen and Chen (2023) showed that 4th grade children could adjust their reading strategies depending on whether they read a single text or multiple texts. Moreover, children with higher vocabulary proficiency were better at adjusting their reading strategies depending on the text they were reading.

In summary, reading skill is an important factor in how selectively children attend to task‐relevant information during reading; children with better reading skills show more rereading and looking back to task‐relevant than to task‐irrelevant text segments. However, previous studies have not distinguished between different types of reading skills, that is technical reading skills (i.e., decoding) or general reading comprehension skills (see, however, Chen et al. 2025, for a study involving vocabulary and reading comprehension). Previous research has established that technical reading skill is especially related to first‐pass reading measures (e.g., de Leeuw et al. 2016a, 2016b). While the effects of reading comprehension have not always been found in either first‐pass reading or look‐backs (e.g., de Leeuw et al. 2016a, 2016b), there is ample evidence that reading comprehension is connected to using strategies that involve looking back in text, which in turn have been connected to integration processes (see Andreou and Gkantaki 2024, for a review). Taken together, this implies that technical reading and reading comprehension skills are manifested in different eye movement patterns.

As suggested by MD‐TRACE (Britt et al. 2022; Rouet and Britt 2011; Rouet et al. 2017), relevance detection and integration are even more crucial in multiple‐text context than single text context for the reader to monitor whether they have accomplished the task set by their task model. Therefore, it is reasonable to assume that the effects of technical reading and reading comprehension are present also in multiple‐text reading. We assume that poor technical readers may recognize relevant segments, which would then elicit longer first‐pass reading times for relevant than for irrelevant segments. As their reading is burdensome, they may not have resources to use advanced reading strategies such as looking back to relevant segments. Furthermore, difficulties in decoding are likely reflected in poorer text synthesis. Better comprehenders, conversely, are likely more skilled in using strategies that facilitate reading comprehension, such as going back to relevant segments of the text. Hence, better reading comprehension should be reflected especially in look‐backs to relevant segments.

Another open question is the relationship between selective processing of text and comprehension outcome. For adults, selective processing of text information during reading results in selective memory for task‐relevant information (e.g., Kaakinen et al. 2002; León et al. 2019; McCrudden et al. 2010). For children, more evidence is needed. Nevertheless, in a study by Ayroles et al. (2021), 5th grade children searched documents for relevant information to answer questions. Participants spent more time on question‐relevant and less time on question‐irrelevant paragraphs when they gave a correct answer to the question. It needs to be noted that Ayroles et al. (2021) examined information search, not text comprehension that would require integration of information across text segments or multiple documents. However, it is likely that when responding to an inquiry task requiring integration of information from multiple sources, readers focus on task‐relevant segments instead of irrelevant parts. To this end, we employed an essay‐writing task and examined the effect of paying attention to task‐relevant segments during reading on the writing task performance.

## 2 Research Questions and Hypotheses

In the present study, we examined children's eye movement patterns as they read multiple expository texts to complete an inquiry task. We were especially interested in seeing how technical reading skill versus reading comprehension modulates the eye movement patterns. We were also interested in whether the eye movement patterns predict the quality of an essay written after reading. We posed two research questions (RQ's):

*Do technical reading skill and reading comprehension skill modulate the effect of task‐relevance on eye movement patterns during reading of multiple texts?*

*Do eye movement measures predict performance in the subsequent essay‐writing task?*

We hypothesized that more skilled readers would pay more attention to task‐relevant than task‐irrelevant text segments, and that this effect would be strongest in the eye movement measures reflecting immediate rereading (in relation to technical reading skill) and later look‐backs (in relation to reading comprehension). Furthermore, we hypothesized that technical reading skill, which affects fluency, will modulate early reading processes (e.g., first‐pass reading), while reading comprehension skill, which involves integration and inferences, will modulate later, more strategic reading (e.g., look‐backs). We also expected that larger relevance effects during reading would be reflected in better quality of the essay written after reading.

## 3 Materials and Methods

### 3.1 Participants

Participants (*N* = 34) were recruited from an elementary school in Salo, Finland. There was no parental permission to combine eye tracking data and independent reading skill measures for ten children. Because of this, 24 children (15 male, 9 female) were included in the analysis, *M*_age_ = 12; 0 years (SD 9 months, range 11; 2–12; 11 years). All spoke Finnish as their first language and had normal or corrected‐to‐normal vision.

### 3.2 Research Ethics Statement

Each participant and their legal guardian gave their consent of participating in the experiment and combining data from the other measurements. The participants received a reflector as a reward. All procedures were performed in accordance with the Declaration of Helsinki. The study was approved in advance by the Ethics Committee for Human Sciences at the University of Turku.

### 3.3 Apparatus

Eye movements were recorded monocularly with EyeLink Portable Duo and EyeLink 1000 (SR Research, Canada) with 500‐Hz sampling rate.^1^ For EyeLink Portable Duo, the texts were presented on a 17.3‐in. Asus ROG G752V laptop (refresh rate of 120 Hz, resolution 1920 × 1080). For EyeLink 1000, the texts were presented on a 24“ BenQ XL2411‐monitor (refresh rate of 120 Hz, resolution 1920 × 1080). A chin‐and‐forehead rest was placed 52 or 76 cm from the screen, respectively. For the essay‐writing task, participants used a regular laptop.

### 3.4 Text Materials

**FIGURE 1:**
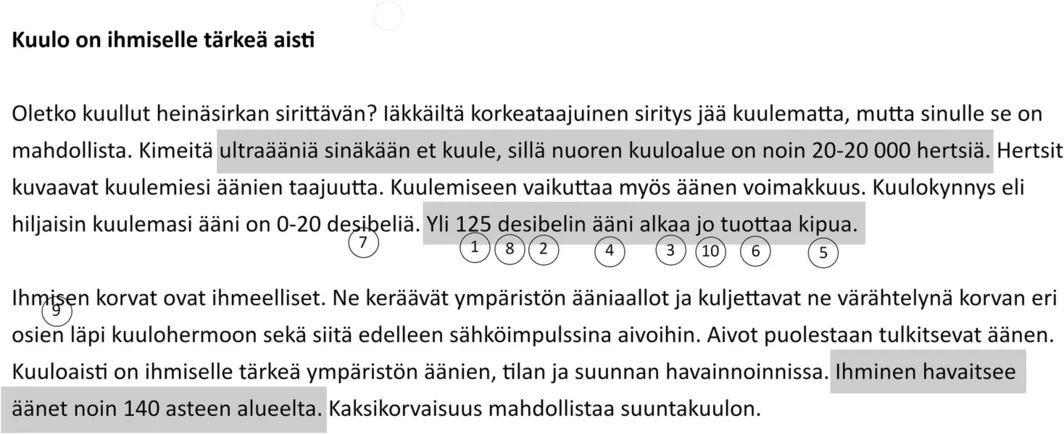
Example of an experimental text “Hearing is an Important Sense for Human” (see Appendix 3 for the English translation). The highlighted areas denote relevant segments. The numbered circles denote fixations, showing an example of an eye movement pattern related to one relevant segment. In this, first‐pass reading duration consists of fixations 1–6; first‐pass rereading duration consists of fixations 4 and 6; look‐back duration consists of fixation 10; as there is fixation 4, probability of a regression within segment is 1; as there is fixation 10, look‐back probability is 1; as there is fixation 7, look‐from probability is 1.

The materials consisted of four age‐appropriate texts written by the research team, designed to resemble science texts found in 5th grade textbooks. Two of the texts concerned hearing (human hearing and dog hearing), and another two concerned eye (human eye and horse eye). Each text was constructed so that it could be understood without the other texts. However, the texts on the same topic (hearing/eye) complemented each other. The texts were on average 98.75 words long (range 98–100 words) and consisted of a headline (3–5 words, not included in the text length) and two paragraphs. Text characteristics are presented in Table 1. Each text was presented on a single screen. The font was proportional Calibri 18 points with 1.5 line spacing. The headline was bolded. An example of a text screen with relevant segments highlighted and a superimposed eye fixation pattern to illustrate the eye movement measures is presented in Figure 1 (English translation in Appendix 1). Each participant read two of the texts (either both eye texts or both hearing texts).

**TABLE 1:** Text characteristics.

Text	Number of words	Number of characters	Readability index
Human hearing	99	700	67.32
Dog hearing	100	709	71.74
Human eye	98	711	57.29
Horse eye	98	638	68.34

*Note:* Number of characters does not include spaces. Readability index is based on Björnsson (1983).

The main task was to complete an inquiry task comparing the differences between human and dog/horse hearing/eye at the end of the study protocol. Texts contained relevant and irrelevant segments with respect to the inquiry task. An example of a relevant text segment is the mention of the human hearing range. It is relevant as the dog hearing text includes a corresponding segment. An example of an irrelevant text segment is a fact concerning how the human brain interprets sound as it does not have a corresponding segment in the dog hearing text. Each text contained information relevant to four core concepts on each topic. For example, for hearing, the relevant concepts were the ability of hearing ultrasound, the threshold of pain, the hearing range, and the auditory perception of the environment.

### 3.5 Essay‐Writing Task

For essay writing, the children used a short version of NEURONE, a simulated WWW environment (González‐Ibáñez et al. 2017). In the essay‐writing task, the participant was first able to review the texts and was asked to highlight 3–5 most task‐relevant segments of a maximum of 10 words from both texts. After highlighting the relevant segments, these segments were presented on the screen and the participant was asked to write an essay to complete the inquiry task. For successful inquiry task completion, the student had to identify relevant text segments dealing with the differences between human and horses' eyes or dogs' hearing, compare these differences, and write a coherent essay.

The essays were coded on four measures: (1) number of relevant words (min = 0, max = 9), (2) relevant concepts concerning human or animal hearing/eye (min = 0, max = 8), (3) coherence based on the logical flow within and between the sentences (min = 0, max = 5), and (4) Task fulfillment that is, the comparison between human and animal hearing/eye (min = 0, max = 4). Two trained coders independently scored a randomized subsample of 30% responses to assess interrater reliability. Quadratic weighted kappas ranged from 0.53 to 0.94 across the matrix dimensions, with a mean kappa of 0.71, indicating substantial agreement (Landis and Koch 1977). As reliability was deemed sufficient, Coder 1 scored the remaining data, and these scores were used for all further analyses. The detailed scoring criteria can be found in Appendix 2.

### 3.6 Measures of Reading Skill and Reading Comprehension

Children completed the technical reading (TL5) and half of the reading comprehension (two expository texts from LY5 for 5th graders, LY6 for 6th graders) subtests of ALLU (Ala‐asteen Lukutesti, Lindeman 1998), a standardized Finnish reading test, in a classroom setting. In the technical reading subtest, children had to recognize and separate words in a word chain that is, string of letters (e.g., therehewho) as quickly as possible. On average, children scored 3.35 (SD 1.40, range 1–6, skewness 0.27) standardized points (scale 1–8, values 4–6 denoting average technical reading skills). The KR20 coefficient of internal consistency of the test is 0.97.

In the reading comprehension subtest, children read two short expository texts and had to answer 12 multiple‐choice questions on the basis of each text. They were allowed to have the texts next to the questions throughout the testing. On average, children scored 4.54 (SD 2.08, range 1–9, skewness 0.37) standardized points (scale 1–9, values 4–6 denoting average reading comprehension). The KR20 coefficients of internal consistency of the tests are 0.62–0.75. Technical reading and reading comprehension correlated at *r* = 0.45.

### 3.7 Procedure

Prior to testing, the children were told that at the end of the testing session, they would have to complete the inquiry task (“What's the difference between human and dog/horse hearing/eye?”) and that they would have to do each subtask with this task in mind.

The eye tracking session started with an internet search page reading task (reported elsewhere). After this, the children were told that they would read two texts, after which they would have to answer the inquiry task (repeated at this stage). They had 4 min to read the first text, and another 4 min to read the second text. The time limits were imposed in order for the whole testing session, including the other tasks, to fit a regular 45‐min class even for the slower readers. They were told they would not need to memorize the texts and that they should read them as they would normally read such texts. Finally, children were told that they would be able to see the texts again prior to the essay‐writing task.

Half of the children read the hearing texts, and the other half read the eye texts. The presentation order of the human and animal texts was counterbalanced between the participants. Before the experiment, a 9‐point calibration procedure was employed. The average calibration error was a maximum of 0.51 degrees. Both texts were preceded by a fixation point at the top‐left corner of the screen. Once the participant fixated the fixation point, the experimenter initiated the text screen. After each text, the participant pressed a mouse button.

After reading the texts, the participants were seated in front of another laptop to use NEURONE (González‐Ibáñez et al. 2017). They were instructed on the highlighting as described above, with the inquiry task being repeated. On the left side of the screen, the children could see the two texts, one at a time. On the right side, the children could select text to make notes and see the notes they had already made. The children were shown how to highlight and save a note with the mouse. After this, the children worked on their notes.

Finally, the children were instructed about the inquiry task, which was repeated once more. They were told that they should use their notes as an aid but that they should not copy/paste text. The target length of the essay was 40–60 words. On the left side of the screen, there was a text box in which the essay was typed in. On the right side, the children could see the notes they had made in the previous phase. The text was automatically saved.

Reading skill and text comprehension tests were completed in a separate session as classroom testing after the eye tracking had taken place.

### 3.8 Data Preparations

The texts were divided into relevant and irrelevant segments, used as areas of interest (AOI) for the analyses of eye movement measures. Segments were identified as relevant based on whether they contained information necessary to perform the inquiry task, either based on concepts (e.g., Hertz, field of vision) or propositions (e.g., explanation of hearing range, how long does it take to get used to dim lighting conditions). Relevant segments were determined by two members of the research team until consensus was reached. The AOIs were based on segments of text consisting of multiple words. The edges of the relevant segments did not always coincide with sentence boundaries. This means that sometimes the relevant segment was only a part of a single sentence (e.g., in the sentence “Hearing sensitivity can also be a problem, as, for example, **fireworks sounding at over 80 decibels cause dogs fear or even pain**.” The bolded area was regarded as a relevant segment and unbolded area as irrelevant). There were two occurrences (but so that each participant only saw one of them) where the relevant segment occupied the end of one sentence, and continued at the beginning of the sentence that followed. Nevertheless, pieces of relevant information belonging to different sentences were treated as separate AOIs (e.g., in the sentence pair “Dog can locate the sound source in a mere sixth of a second and **hear very high frequencies from a 360 degree area surrounding it**. *The frequency band of the dog's hearing range is 70–100,000 hertz, meaning they also hear very high ultra sounds*.” In which the bolded area was regarded as one relevant segment and the italicized area as another relevant segment). The relevant text segments were spread over the whole text. The first sentence was never a relevant segment. On average, the relevant AOIs consisted of 9.50 words (SD 3.43, range 4–19). The remaining text segments were considered as irrelevant. As for the relevant segments, if an irrelevant segment extended over a sentence boundary, the parts in different sentences were treated as separate AOIs. On average, the irrelevant AOIs consisted of 7.55 words (SD 4.64, range 1–19).

For the eye tracking data, the following automatized clean‐up procedures were used: Fixations closer to each other than 0.5 degrees were merged; remaining fixations shorter than 50 ms were deleted; and fixations immediately before or after a blink were deleted. All of the remaining fixations were treated as separate fixations. Fixation patterns were visually inspected and hand‐corrected to be on lines if they clearly fell below them. If a fixation pattern was not clear but was judged to look like reading, its fixations were left on their original places. If a fixation pattern did not resemble reading (e.g., a sequence of fixations darting around the text instead of following the usual left‐to‐right pattern), its fixations were deleted. For the beginning of reading, if there was a single fixation not on headline or first sentence, or more than one fixation not on headline or first sentence but so that they were all on different segments, they were deleted. Finally, if during first‐pass reading of any segment, there was a single fixation in an upcoming area of interest (i.e., the AOI was exited to an upcoming AOI where a single fixation was made before returning to the previously mentioned AOI), it was deleted. The rationale for this was that it can be argued that a single fixation to an upcoming AOI followed by an immediate regression back to the previously mentioned AOI practically indicates that the first‐pass processing of said AOI is still ongoing.

### 3.9 Statistical Analyses

The data were analyzed with (generalized) linear‐mixed effects models (Baayen et al. 2008; lme4 package, version 1.1–35.4, Bates et al. 2015) and linear regression (basic R Stats package) using R statistical software (version 4.3.2; R Core Team 2023). The plots have been created with ggplot2 (version 3.5.1; Wickham 2016). As one of the participants was not present for the technical reading skill classroom testing, their data was discarded from the analyses.

For RQ1, we analyzed several duration and probability measures. For duration, the dependent variables were first‐pass reading duration (summed duration of all fixations in the AOI before exiting the segment for the first time), first‐pass rereading duration (summed duration of all regressive fixations in the AOI before exiting the segment for the first time), and look‐back time (Hyönä et al. 2003; summed duration of all fixations made on the AOI after exiting it for the first time). As the AOIs were of different sizes, the duration measures were transformed to ms/character scale. The duration measures were then log transformed to correct for skewness. For probability, the dependent variables were probability of making a regression within a segment during first‐pass reading, probability of look‐back (making a look‐back landing in that particular AOI), and probability of look‐from (making a look‐back leaving that particular AOI). Each probability variable was coded as 1 for event and 0 for no event.

Separate models were run for each dependent variable. For each model, the critical independent variable was relevance of the segment (relevant/irrelevant). Relevance was deviation‐coded (relevant 0.5, irrelevant −0.5). We also used technical reading skill and Reading comprehension as independent variables (*z*‐transformed). These reading skill measures were continuous, but for the sake of clarity, we will discuss them in terms of better and worse readers (i.e., those that scored higher or lower in said measure, respectively). For the reading time variables, zeros (e.g., an AOI having a First‐pass reading duration of 0) were treated as missing values, resulting in conditional reading time measures. This amounted to 4.86% missing values for First‐pass reading duration, 33.25% for First‐pass rereading duration, and 54.30% for Look‐back duration. To answer RQ1, we included the interactions of relevance and technical reading as well as relevance and reading comprehension. We attempted to fit the maximal random structure (Barr et al. 2013). Due to convergence issues, to find the model with the maximal converging random structure, one random slope at a time was removed from the model. This was done for all possible random slope combinations. The model with the most complex random structure that converged was selected as the final model. For the sake of brevity, the final models are reported in Appendix 3. The interactions are interpreted on basis of graphs.

For RQ2, we predicted essay scores on the basis of eye movement indices and reading skill measures. The eye movement indices were extracted by running a separate model for every measure (first‐pass reading duration, probability of making a regression within a segment during first‐pass reading, probability of look‐back, and probability of look‐from) for each individual participant (Kliegl et al. 2011). For the duration measures, only first‐pass reading duration was used due to the abundance of zeros for the regressive durations. In each model, deviation‐coded relevance (relevant 0.5, irrelevant −0.5) was used as the sole predictor. From the resulting models, we extracted intercept and relevance coefficients for each variable for each participant. The intercept coefficients were *z*‐transformed. In order not to overfit, we wanted to have only a small set of predictors. As many of the coefficients were highly correlated (see Table 2), we selected intercept and relevance coefficients of first‐pass reading duration and probability of a look‐back to have indices of early reading and later reading, respectively. First‐pass reading duration was selected as it reflects the initial time spent processing the segment. As for later processing, the ideal choice would have been look‐back duration. However, as for this measure, there were more than 50% zero values, we selected the probability of look‐back as our late processing index.

**TABLE 2:** Correlations between the coefficients extracted for individual participants.

	FD (int)	FD (rel)	*p*FR (int)	*p*FR (rel)	*p*LB (int)	*p*LB (rel)	*p*LF (int)	*p*LF (rel)
FD (int)	1							
FD (rel)	0.10	1						
*p*FR (int)	0.56	0.11	1					
*p*FR (rel)	0.23	0.31	0.71	1				
*p*LB (int)	−0.21	0.13	0.06	0.11	1			
*p*LB (rel)	−0.03	−0.26	−0.25	−0.35	−0.03	1		
*p*LF (int)	−0.11	0.17	0.08	0.17	0.84	−0.31	1	
*p*LF (rel)	−0.02	−0.10	0.03	0.04	0.23	−0.01	0.23	1

Abbreviations: FD, first‐pass duration; int., intercept; *p*FR, probability of first‐pass regression; *p*LB, probability of look‐back; *p*LF, probability of look‐from; rel, relevance.

Next, we ran regression models with technical reading, reading comprehension, first‐pass reading intercept and relevance coefficient, and probability of a look‐back intercept and relevance coefficient as independent variables. The synthesis scores used as dependent variables were number of relevant words, conceptual science content, and coherence. As the majority of participants performed close to floor level in task fulfillment, it was not analyzed. The *t*/*z* values reported are the statistics for the fixed effects in the mixed effects/regression models. For the sake of clarity, we will only report reliable effects and interactions apart from the effect of relevance, for which a standardized *β* or OR is provided for every measure.

Anonymized data and R script used for analyses can be found at https://osf.io/y5vh4/.

## 4 Results

### 4.1 Do Technical Reading Skill and Reading Comprehension Skill Modulate the Effect of Task‐Relevance on Eye Movement Patterns During Reading of Multiple Texts?

The non‐transformed descriptive statistics for the dependent variables are presented in Table 3. The final models are presented in Appendix 3 as Tables A3, A4, A5, A6, A7, A8.

**TABLE 3:** The descriptive statistics for the eye movement variables in relevant and irrelevant segments.

	Relevant		Irrelevant	
	*M*	SD	*M*	SD
First‐pass reading duration	52.03	33.40	49.11	32.83
First‐pass rereading duration	14.69	20.87	10.92	15.23
Look‐back duration	19.19	35.96	15.36	35.89
Probability of a regression within segment	0.76	0.43	0.67	0.47
Look‐back probability	0.43	0.50	0.43	0.50
Look‐from probability	0.52	0.50	0.43	0.50

*Note:* Durations are in ms/character.

*First‐pass reading duration*. Relevant segments were read for longer than irrelevant segments, *β =* 0.16, *t* = 2.12. Furthermore, readers with better technical reading skill had shorter first‐pass reading durations, *t* = −2.32. There were no reliable interactions, all *t* < 1.

*First‐pass rereading duration*. The effect of relevance (*β =* 0.08) nor other effects reached statistical significance, all *t*s ≤ 1.4.

*Look‐back duration*. Better comprehenders had shorter look‐back durations, *t* = −3.04. Furthermore, readers with better technical reading skill had longer look‐back durations, *t* = 2.76. There were no reliable interactions or a relevance effect (*β =* 0.10), all *t*s < 1.

**FIGURE 2:**
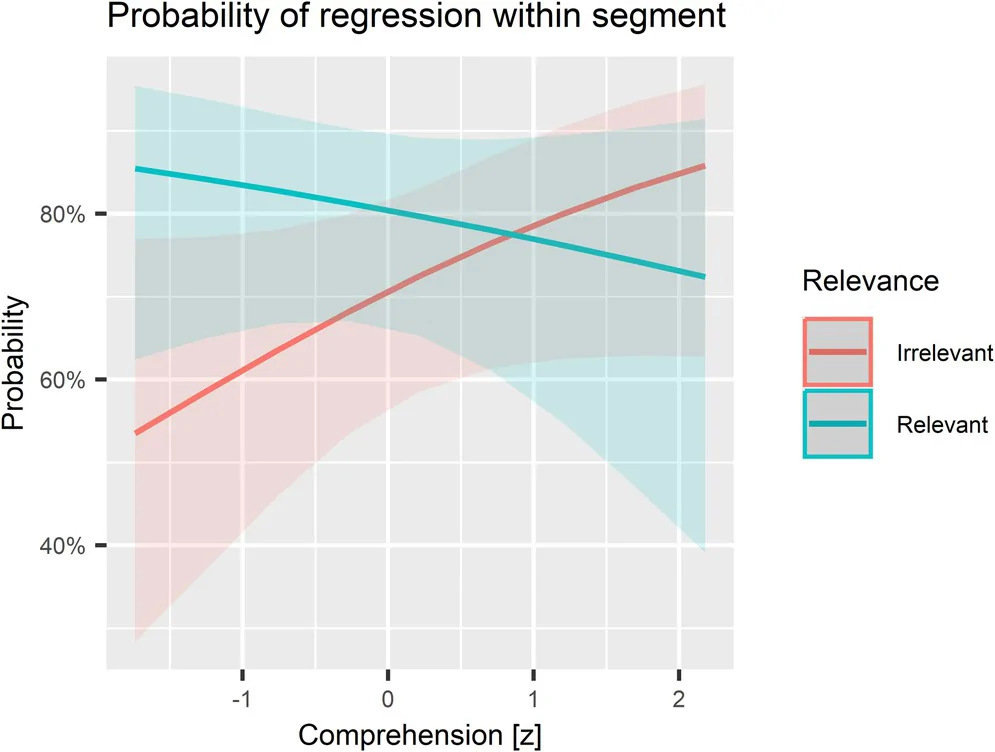
The effect of comprehension on the probability of making a regression within segment for relevant and irrelevant segments. Shaded areas denote 95% CIs.

*Probability of making a regression within a segment during first‐pass reading*. There was no reliable effect of relevance (OR = 1.70), but there was an interaction between relevance and comprehension, *z* = −2.39, depicted in Figure 2. Weakest comprehenders showed a higher probability of making a regression within relevant than irrelevant segments. For better comprehenders, this difference disappeared.

**FIGURE 3:**
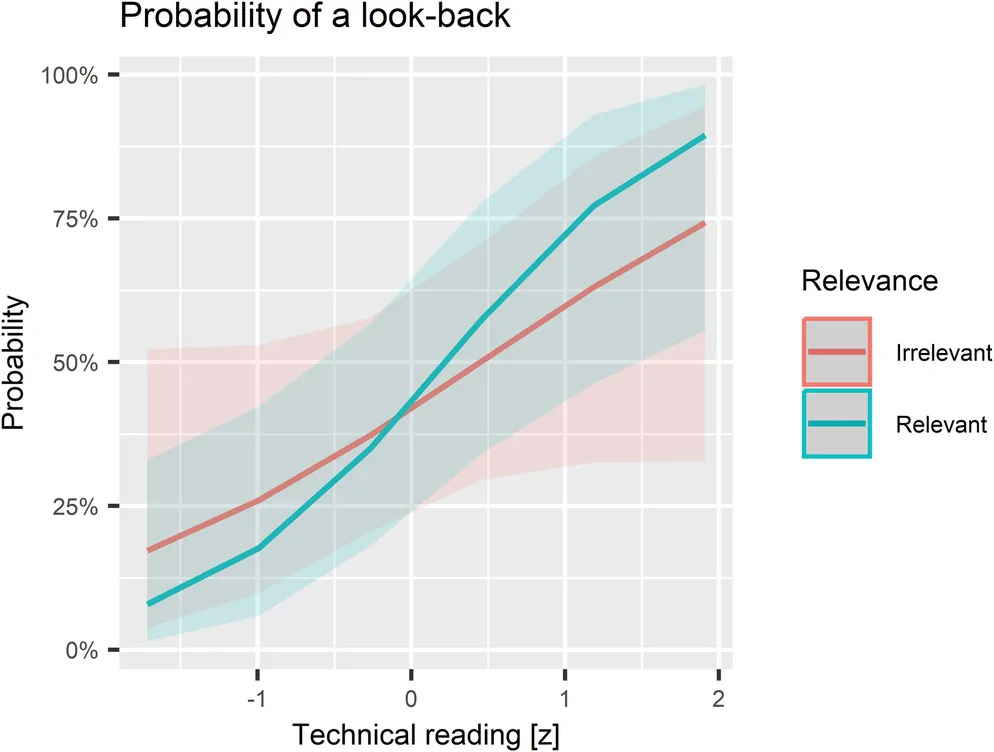
The effect of technical reading skill on the probability of making a look‐back for relevant and irrelevant segments. Shaded areas denote 95% CIs.

*Probability of a look‐back*. While there was no reliable effect of relevance (OR = 1.05), there was an interaction between relevance and technical reading, *z* = 1.97, depicted in Figure 3. Overall, readers with better technical reading skills had a higher probability of making a look‐back in both relevant and irrelevant segments than those with weaker technical reading skills. While for the readers with better technical reading skills the probability of making a look‐back was numerically higher for relevant than irrelevant segments, for readers with weaker technical reading skills this effect was reversed.

**FIGURE 4:**
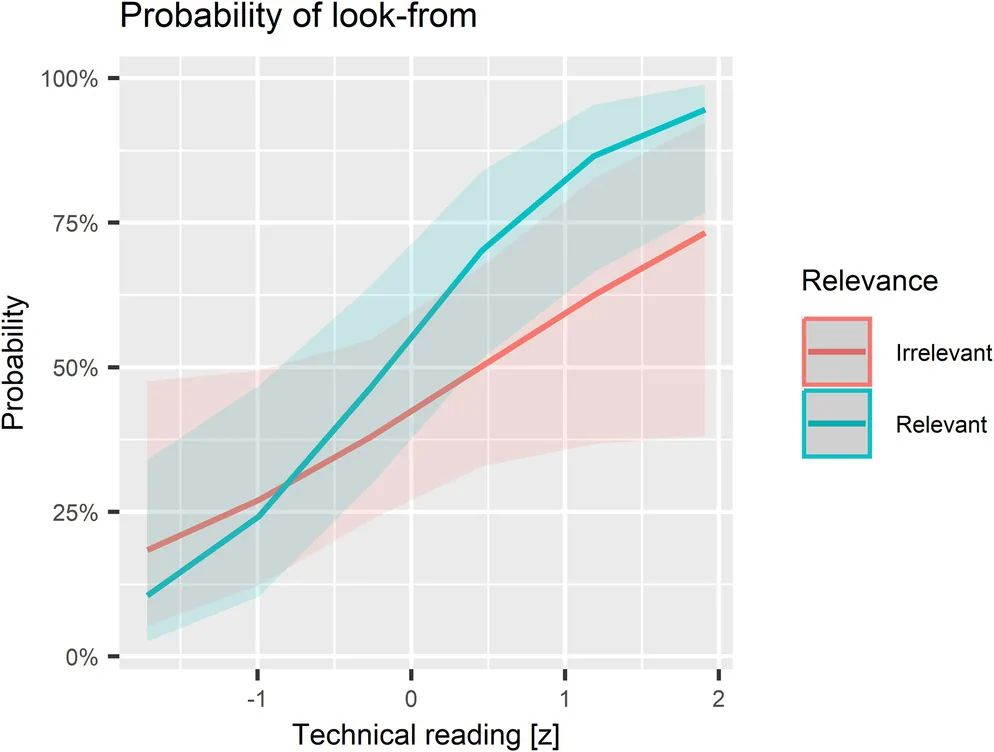
The effect of technical reading skill on the probability of making a look‐from for relevant and irrelevant segments. Shaded areas denote 95% CIs.

*Probability of a look‐from*. There was no reliable effect of relevance (OR = 1.71) but there was an interaction between relevance and technical reading skill, *z* = 2.53, depicted in Figure 4. Readers with better technical reading skills had a higher probability of making a look‐from than those with weaker skills. While readers with better technical reading skills had a higher probability of initiating a look‐from from a relevant than an irrelevant segment, for weaker technical readers there was practically no effect of relevance. Finally, there was a main effect of comprehension with better comprehenders having a lower probability of making a look‐from, *z* = −2.34.

### 4.2 Do Eye Movement Measures Predict Performance in the Subsequent Essay‐Writing Task?

The descriptive statistics for the dependent variables (including task fulfillment) and their correlations with technical reading and reading comprehension are presented in Table 4. The essays were on average 63.67 words long (SD 25.62, range 42–154). The final models for all essay‐writing task measures are presented in Appendix 3 as Tables A9, A10, A11. None of the effects were reliable, all *t*s < 2. For relevant words in the essay, neither first‐pass reading duration (*β =* 0.37) nor probability of a look‐back (*β =* −0.01) were reliable predictors. Also, for the conceptual science content, first‐pass reading duration (*β =* −0.16) or probability of a look‐back (*β =* −0.08) were not reliable predictors. The same was true for coherence: neither first‐pass reading duration (*β =* 0.08) nor Probability of a look‐back (*β =* 0.16) predicted essay coherence. Of note, for Conceptual science content and Coherence, the adjusted *R* ^2^s were negative, providing further statistical evidence that the indices were not predictive of essay quality.

**TABLE 4:** The descriptive statistics and correlations with technical reading and reading comprehension for the essay writing scores.

	*M*	SD	Min	Max	Technical reading	Reading comprehension
Number of relevant words	5.65	1.31	3	8	−0.02	0.30
Conceptual science content	3.98	1.75	0	7	0.05	0.14
Coherence	2.42	1.35	1	5	0.15	0.40
Task fulfillment	1.69	1.11	0	4	−0.13	−0.03

## 5 Discussion

We were interested in the effect of relevance on multiple text reading of 5th and 6th grade Finnish children. Participants were presented with an inquiry task and two expository texts related to the task. The texts contained both task‐relevant and task‐irrelevant text segments. At the end of the experiment, participants completed an essay‐writing task. Task‐relevant segments were read longer than task‐irrelevant segments during first‐pass reading. Moreover, weaker comprehenders were less likely to regress within an irrelevant segment. Better technical reading was associated with shorter first‐pass and longer rereading durations. Furthermore, the relevance effect was more pronounced for better technical readers with respect to rereading. As for the essay‐writing task, the eye tracking indices did not predict performance in it and overall no reliable effects were found for the essay‐writing task.

### 5.1 Effects of Task Relevance on Eye Movement Patterns

We hypothesized that more skilled readers would pay more attention to task‐relevant than to task‐irrelevant text segments and that this effect would be strongest in the eye movement measures reflecting immediate rereading and subsequent look‐backs. We found only partial support for this hypothesis.

Technical reading skill did not modulate the effect of relevance during first‐pass reading, even though better technical readers had shorter first‐pass reading durations overall. This trivial finding of reading skill is consistent with previous studies (e.g., de Leeuw et al. 2016a; Häikiö et al. 2018; Hautala et al. 2019; van den Broek et al. 2009). While the modulating effect of reading ability on relevance was not observed in duration measures, it was observed in the probability of making a look‐from or a look‐back. While better technical readers had a higher likelihood of look‐backs overall, this effect was more pronounced for relevant segments. This finding is consistent with our hypothesis. This pattern of results demonstrates the need to integrate relevant information with earlier text, even though better technical readers tended to go back and read most of the text again anyway. For poorer technical readers, the probabilities associated with look‐backs did not differ between relevant and irrelevant segments, showing that their direction of later reading was not efficient. These findings are in line with those of van den Broek et al. (2009), who showed that skilled child readers focused their look‐backs on relevant segments, whereas less skilled readers did not systematically direct their look‐backs.

The finding that poorer technical readers were less likely to make look‐backs overall contradicts the results of previous studies, which either showed longer look‐back durations for poorer decoders (Häikiö et al. 2018; Hautala et al. 2019) or no effect of decoding (de Leeuw et al. 2016a). This is likely due to our specific task. Our participants saw the texts again before completing the essay‐writing task, in contrast to previous studies in which participants had to answer comprehension questions by memory. It is likely that because decoding was more effortful for poorer technical readers, they devoted their processing resources to first‐pass reading, knowing that they could read the texts again at a later stage.

As for the effects related to reading comprehension skill, weaker comprehenders were less likely to make a regression within irrelevant segments than within relevant segments during first‐pass reading, whereas better comprehenders made regressions within both relevant and irrelevant segments at approximately the same, relatively high rate. This suggests that better comprehenders developed a strategy of non‐selective first‐pass reading, knowing that they can return to important segments of the text. While weaker comprehenders recognize the relevance of the segment, it is hard to say whether the lower probability of a regression within the segment is due to a decision of focusing their initial efforts on reading relevant parts more thoroughly or getting disengaged while reading irrelevant segments.

Against our hypothesis, there were no relevance effects for look‐backs in relation to comprehension, contradicting van der Schoot et al. (2008), who showed that better comprehenders in grades five and six spent relatively more time in making look‐backs to relevant words. However, our study used relevant and irrelevant segments, whereas van der Schoot et al. used single (ir)relevant target words within texts. It may be that when task relevance is based on a single word rather than longer segments, a single word plays a relatively larger role in text comprehension, necessitating a re‐examination of whether it makes the entire segment relevant. Nevertheless, our findings are in line with those of Chen et al. (2025) who did not find an effect of reading comprehension in their entropy measure for second‐pass reading in the condition in which the children read for general comprehension. Interestingly, better comprehenders had shorter overall look‐back durations, suggesting that they processed the information efficiently during the first‐pass reading.

Overall, we observed the relevance effect in first‐pass reading, reflecting that our participants, regardless of their reading skill, recognized relevant segments and spent more time reading them. This finding is consistent with that of van der Schoot et al. (2008), who observed that 5th and 6th grade readers had longer first‐pass fixations on relevant words, regardless of reading skill. Furthermore, our participants went more often back and forth between relevant than irrelevant segments, showing sensitivity to our relevance manipulation. This relevance effect contradicts the finding of Schumacher et al. (1983), who did not observe that the look‐backs of 5th grade children would be directed to task‐relevant segments. However, it is consistent with the finding of Hautala et al. (2019), who witnessed 6th grade children making more look‐backs to task‐relevant segments of the text. Notably, the language of the children in the Hautala et al. study was Finnish, as it was for our children. Since children reading in Finnish, a language with transparent orthography, have been proficient readers for a longer time than those in English, a language with opaque orthography (see Seymour et al. 2003), they may possess more resources for building text representations and, hence, locate task‐relevant segments better.

In the present study, we used Look‐back measures as an indicator of relevance detection, as has been done in previous studies (e.g., Hautala et al. 2019; Rouet et al. 2001). It can also be argued that Look‐back behavior is indicative of integration attempts (e.g., Kaakinen and Hyönä 2005). As we did not include a task in which the task model would have been explicitly measured, such as think‐aloud (e.g., Kaakinen and Hyönä 2005), we cannot disentangle between these two accounts. At any rate, we believe these measures signal both relevance detection and integration attempts.

One point to note is that our task required inter‐text integration. What was task‐relevant was not obvious when reading the first of the two texts, because at that point there was no information about which parts of the text would contain information relevant for the comparisons. This may be a trivial reason for not seeing all of the expected effects. The reason we selected sequential presentation of texts was two‐fold. First, this was done to mimic real‐life reading in which it is quite usual that people read texts in a sequential order instead of jumping back and forth between them, at least for the first time they encounter the text. This has also been the usual presentation mode in previous eye tracking studies in which several texts have been presented (e.g., Kaakinen and Hyönä 2005; however, see Chen et al. 2025, for an example of simultaneous presentation of texts). Second, our design in which the participants could not go back to a previously read text during the eye tracking ensures that our Look‐back measures are purer in indicating immediate integration processes and comparable across participants, as all participants saw the texts in the same order.

### 5.2 Effects of Reading on Essay‐Writing Task

For our second hypothesis, we expected that larger relevance effects during reading would be reflected in better scores on various aspects of the post‐reading essay‐writing task. However, we did not observe any reliable effects in the essay‐writing task, further underscored by the negative adjusted *R* ^2^s in concepts and coherence. Overall, participants had relatively high scores of relevant words and concepts but lower scores on coherence and task fulfillment.

As for readers of different reading skills, we did not find any statistically significant differences with respect to their essay‐writing scores (van den Broek 2010; van den Broek et al. 2002, 2011). The coherence and task fulfillment scores suggest that the standards of coherence set by the children were not sufficient to complete the task well. As mentioned above, weaker comprehenders seemed to adjust their reading according to the standards of coherence as defined by the reading task. However, this did not lead to better essay‐writing scores. Moreover, better comprehenders made shorter look‐backs overall and showed no relevance effects during first‐pass reading. It may be that they understood the gist of the text during first‐pass reading and had no need to allocate resources to more thorough processing.

The null findings regarding the link between the observed process data (i.e., eye tracking indices) and final outcome measures (i.e., essay‐writing scores) are likely due to the nature of our task in which the children could revisit the texts during the essay‐writing task. Getting to see the texts again may have shifted the task demands from being memory‐based integration to more text‐centered synthesis process. Furthermore, the time constraints during the reading may have affected the performance, at least for slower readers. It may be the case that they have not had time to fully process the texts, or may have had to resort to using a different type of strategy than they would have otherwise used, for instance processing the texts more thoroughly during first‐pass reading than they would have preferred. However, suboptimal reading strategy should also be reflected in lower essay scores and relationship between the eye tracking indices and essay scores. At any rate, the future studies should examine the process‐product gap witnessed in our study. This could be done by, for instance, employing a memory‐based essay‐writing task in which the participants do not have the opportunity to revisit the texts, or measuring the eye movement patterns of the participants while they are completing the essay‐writing task.

There is an important caveat here: the essay‐writing task is not a pure measure of comprehension as it involves many processes. A child might have built a coherent mental model of the topic but struggle in the actual writing task. As we did not explicitly measure the task model with, for instance, explicitly asking what the children think is the goal of the task, we cannot disentangle between these two accounts. However, we aimed at ecological validity as this type of task is often used in school. Moreover, the independently measured reading comprehension correlated with coherence at *r* = 0.40. While this effect failed to reach significance and there was no effect of task fulfillment, for instance, this correlation indicates that better comprehenders indeed performed better in this writing task. Furthermore, the essay‐writing task is regularly used as a measure in multiple text integration (see Primor and Katzir 2018, for a review). It can be said that writing an essay is a generally accepted and common task to measure multiple text comprehension and, hence, the coherence of the mental model.

## 6 Limitations

The biggest limitation of the current study is the small sample size, due to our study being carried out during the COVID‐19 pandemic. This has led to low statistical power, which may have obscured the full pattern of results. That said, the main findings are solid, indicating that we did have enough power to detect interaction between relevance and reading skill measures. At any rate, future studies should replicate these findings with larger samples. While we found relevance effects in the reading task, they did not carry over to the essay‐writing task. Although this may reflect the fact that 5th and 6th graders do not set their standards of coherence high enough to perform adequately in an inquiry task, it may also be that our specific task was simply not conducive to finding such effects. Because the relevance of one segment depended on the material presented in the other text, our participants may have had difficulty setting adequate standards of coherence from the first time they read the texts. In addition, the task instructions emphasized that the texts would be presented later, which may have led participants to adopt a familiarization strategy during the eye tracking phase rather than reading more strategically. Next, we did not measure children's prior knowledge on the topic. However, we had carefully chosen the topics so that they had not been covered in the school curriculum. While it is possible that for some children the topic was familiar, it is highly unlikely it was familiar to the majority of participants. Finally, we did not explicitly measure children's task model. Therefore, we cannot be sure whether the null results are indicative of the failure of task model, difficulties in the essay‐writing task, or simply the way our task was constructed. These aspects should be investigated in future studies, which could also benefit from inclusion of other types of measures that can be combined with eye tracking, such as entropy as used by Chen et al. (2025) or engagement as measured by postural swaying in the study of Ballenghein and Lachaud (2025).

## 7 Conclusion

Finnish 5th and 6th grade readers recognize what is relevant to the task‐at‐hand and adjust their reading accordingly, based on their reading skills. While better technical reading skill was connected to more selective later look‐backs, less skilled comprehenders engaged in more first‐pass rereading of relevant segments. Despite children's sensitivity to the task relevance during reading, they did not seem to form a coherent memory representation of the relevant text contents. One possible reason for this is that the inquiry task used required integrating information across two texts. Since integrating information across multiple texts is an important part of text comprehension in modern society, our findings suggest that primary school children need to be better taught to monitor their reading comprehension in relation to the task at hand.

## Author Contributions

**Tuomo Häikiö:** conceptualization, data curation, formal analysis, investigation, methodology, software, writing – original draft, writing – review and editing. **Oksana Kanerva:** conceptualization, data curation, investigation, writing – review and editing. **Norbert Erdmann:** conceptualization, methodology, software, writing – review and editing. **Mirjamaija Mikkilä‐Erdmann:** conceptualization, funding acquisition, writing – review and editing. **Johanna K. Kaakinen:** conceptualization, funding acquisition, methodology, writing – review and editing.

## Funding

This study was funded by the Strategic Research Council/Academy of Finland grant numbers 335233 and 358271.

## Ethics Statement

All procedures were performed in accordance with the Declaration of Helsinki. The study was approved in advance by the Ethics Committee for Human Sciences at the University of Turku.

## Conflicts of Interest

The authors declare no conflicts of interest.

## Data Availability

Anonymized data and R script used for analyses can be found at https://osf.io/y5vh4/.

## Endnotes

^1^
Two eye trackers were used as we needed to finish testing before possible Covid‐19 lockdown. When we used the tracker type as a covariate in the statistical analyses, it did not change the pattern of results.

## Appendix 1 English Translation of the Text Presented in Figure 1

Hearing is an important sense for humans.

Have you heard a grasshopper chirping? High‐frequency chirping is unheard by the elderly, but it is possible for you. You also will not hear harsh ultrasounds, because a young person's hearing range is around 20–20,000 hertz. Hertz describes the frequency of the sounds you hear. Hearing is also affected by the volume of the sound. The hearing threshold that is, the quietest sound you hear is 0–20 dB. A sound above 125 dB already starts to cause pain.

Human ears are amazing. They collect the sound waves of the environment and transport them as vibrations through different parts of the ear to the auditory nerve and from there as an electrical impulse to the brain. The brain, in its turn, interprets the sound.

The sense of hearing is important for humans in perceiving environmental sounds, space, and direction. Humans perceive sounds from a range of about 140 degrees. Having two ears enables directional hearing.

## Appendix 2 Points Criteria for the Essay Writing Task

*Number of relevant words used in the explanation concerning human or animal hearing/eye*. One point was awarded for each relevant word mentioned in the texts for both the hearing and eye tasks. The maximum score was 9. For the hearing inquiry task, the relevant words (translated from Finnish) were human, dog, ear, hearing (mentioning “to hear” instead was awarded with 0.5 points), hearing area, frequency, hertz, ultrasound, decibel. For the eye inquiry task, the relevant words (translated from Finnish) were human, horse, eye, vision (mentioning “to see” instead was awarded with 0.5 points), field of view, dim/dark vision, three‐dimensional, accommodation, adaptation.

*Relevant concepts concerning human or animal hearing/eye*. One point was awarded for each relevant concept mentioned in the texts for both the hearing and eye tasks. Furthermore, separate points were awarded for explaining the concept for human and dog/horse each, i.e., explaining the frequency range of hearing for human resulted in one point, and explaining it for dog resulted in one point. As there were four concepts per topic, the maximum score was 8. For the hearing inquiry task, the concepts were ultrasound, frequency, hearing area, and hearing threshold. For the eye inquiry task, the concepts were field of view, three‐dimensionality, adaptation, and accommodation.

*Coherence based on the logical flow within and between the sentences*. Points were awarded for coherence according to the criteria presented in Table A1.

*Task fulfillment that is, the comparison between human and animal hearing/eye*. Points were awarded for task fulfillment according to the criteria presented in Table A2.

**TABLE A1:** Points awarded for coherence in the essay writing task.

Evaluation criteria	Points
No sentences	0
No linked sentences	1
Partial coherence within sentences	2
Mainly coherent within sentences	3
Mainly coherent within sentences and partially coherent flow between sentences	4
Coherent flow within and between sentences	5

**TABLE A2:** Points awarded for task fulfillment in the essay writing task.

Evaluation criteria	Points
No comparison	0
Comparisons only listed, not linked	1
2 linked comparisons	2
3 linked comparisons	3
4 linked comparisons	4

## Appendix 3

**TABLE A3:** The final lmer model for first‐pass reading duration (First‐PassReadingDuration ~1 + Relevance × [Comprehension.z + TechnicalReading.z] + [1|participant] + [1|segment]).

Predictors	Estimate	SE	CI	*t*	*p*
Intercept	**3.74**	**0.07**	**3.61 to 3.88**	**54.12**	**< 0.001**
Relevance [relevant]	**0.09**	**0.04**	**0.01 to 0.18**	**2.12**	**0.035**
Comprehension	0.09	0.08	−0.06 to −0.24	1.22	0.222
Technical reading	**−0.18**	**0.08**	**−0.33 to −0.03**	**−2.32**	**0.021**
Relevance [relevant] × comprehension	−0.01	0.05	−0.10 to 0.09	−0.13	0.893
Relevance [relevant] × technical reading	−0.01	0.05	−0.10 to 0.09	−0.12	0.908
Random effects					
*σ* ^2^	0.23				
*τ*_00participant_	0.10				
ICC	0.30				
*N*_participant_	23				
Observations	518				
Marginal *R* ^2^/Conditional *R* ^2^	0.081/0.359				

*Note:* Duration is in ms/character that has been log‐transformed. Technical reading and comprehension have been *z*‐transformed. Bolding denotes coefficients with *p* < 0.05.

**TABLE A4:** The final lmer model for first‐pass rereading duration (First‐PassRereadingDuration ~1 + Relevance × [Comprehension.z + TechnicalReading.z] + [Relevance|participant] + [Comprehension.z|segment]).

Predictor	Estimate	SE	CI	*t*	*p*
Intercept	**2.44**	**0.08**	**2.28 to 2.61**	**29.14**	**< 0.001**
Relevance [relevant]	0.07	0.11	−0.16 to 0.29	0.58	0.560
Comprehension	−0.08	0.08	−0.24 to 0.08	−0.93	0.351
Technical reading	−0.09	0.08	−0.26 to 0.07	−1.13	0.258
Relevance [relevant] × comprehension	−0.00	0.09	−0.18 to 0.18	−0.03	0.979
Relevance [relevant] × technical reading	0.12	0.09	−0.05 to 0.30	1.40	0.163
Random effects					
*σ* ^2^	0.46				
*τ*_00sentence_	0.07				
*τ*_00participant_	0.09				
*τ*_11segment.comprehension.z_	0.01				
*ρ*_01segment_	−0.13				
ICC	0.28				
*N*_participant_	23				
*N*_sentence_	46				
Observations	356				
Marginal *R* ^2^/Conditional *R* ^2^	0.047/0.310				

*Note:* Duration is in ms/character that has been log‐transformed. Technical reading and comprehension have been *z*‐transformed. Bolding denotes coefficients with *p* < 0.05.

**TABLE A5:** The final lmer model for look‐back duration (Look‐backDuration ~1 + Relevance × [Comprehension.z + TechnicalReading.z] + [1|participant] + [1|segment]).

Predictor	Estimate	SE	CI	*t*	*p*
Intercept	**2.76**	**0.14**	**2.48 to 3.04**	**19.31**	**< 0.001**
Relevance [relevant]	0.09	0.17	−0.24 to 0.43	0.53	0.593
Comprehension	**−0.43**	**0.14**	**−0.72 to −0.15**	**−3.04**	0.**003**
Technical reading	**0.41**	**0.15**	**0.12 to 0.70**	**2.76**	0.**006**
Relevance [relevant] × comprehension	−0.06	0.14	−0.33 to 0.21	−0.47	0.640
Relevance [relevant] × technical reading	0.13	0.14	−0.14 to 0.40	0.94	0.346
Random effects					
*σ* ^2^	0.74				
*τ*_00sentence_	0.13				
*τ*_00participant_	0.27				
ICC	0.35				
*N*_participant_	22				
*N*_sentence_	47				
Observations	249				
Marginal *R* ^2^/Conditional *R* ^2^	0.130/0.435				

*Note:* Duration is in ms/character that has been log‐transformed. Technical reading and comprehension have been *z*‐transformed. Bolding denotes coefficients with *p* < 0.05.

**TABLE A6:** The final glmer model for the probability of a regression within segment (ProbabilityOfARegressionWithinSegment ~1 + Relevance × [Comprehension.z + TechnicalReading.z] + [1|participant] + [1|segment]).

Predictor	OR	SE	CI	*z*	*p*
Intercept	**3.14**	**0.88**	**1.81 to 5.45**	**4.08**	**< 0.001**
Relevance [relevant]	1.70	0.70	0.76 to 3.83	1.29	0.197
Comprehension	1.11	0.27	0.69 to 1.80	0.44	0.657
Technical reading	0.78	0.20	0.48 to 1.28	−0.99	0.321
Relevance [relevant] × comprehension	**0.53**	**0.14**	**0.32 to 0.89**	**−2.39**	0.**017**
Relevance [relevant] × technical reading	1.57	0.42	0.92 to 2.67	1.67	0.095
Random effects					
*σ* ^2^	3.29				
*τ*_00sentence_	1.23				
*τ*_00participant_	0.80				
ICC	0.38				
*N*_participant_	23				
*N*_sentence_	47				
Observations	518				
Marginal *R* ^2^/Conditional *R* ^2^	0.039/0.405				

*Note:* Technical reading and comprehension have been *z*‐transformed. Bolding denotes coefficients with *p* < 0.05.

**TABLE A7:** The final glmer model for the probability of a look‐back (ProbabilityOfALook‐back ~1 + Relevance × [Comprehension.z + TechnicalReading.z] + [1|participant] + [1|segment]).

Predictor	OR	SE	CI	*z*	*p*
Intercept	0.76	0.29	0.35 to 1.62	−0.72	0.471
Relevance [relevant]	1.05	0.35	0.54 to 2.02	0.14	0.889
Comprehension	0.49	0.20	0.22 to 1.08	−1.76	0.078
Technical reading	**2.71**	**1.15**	**1.18 to 6.24**	**2.34**	0.**019**
Relevance [relevant] × comprehension	0.91	0.24	0.54 to 1.54	−0.34	0.737
Relevance [relevant] × technical reading	**1.72**	**0.47**	**1.00 to 2.95**	**1.97**	0.**049**
Random effects					
*σ* ^2^	3.29				
*τ*_00sentence_	0.61				
*τ*_00participant_	2.77				
ICC	0.51				
*N*_participant_	23				
*N*_sentence_	47				
Observations	518				
Marginal *R* ^2^/Conditional *R* ^2^	0.112/0.561				

*Note:* Technical reading and comprehension have been *z*‐transformed. Bolding denotes coefficients with *p* < 0.05.

**TABLE A8:** The final glmer model for the probability of a look‐from (ProbabilityOfALook‐from ~1 + Relevance × [Comprehension.z + TechnicalReading.z] + [1|participant] + [Comprehension.z|segment]).

Predictor	OR	SE	CI	*z*	*p*
Intercept	0.99	0.32	0.53 to 1.84	−0.04	0.965
Relevance [relevant]	**1.71**	**0.46**	**1.01 to 2.88**	**2.01**	**0.045**
Comprehension	**0.44**	**0.15**	**0.22 to 0.88**	**−2.34**	**0.020**
Technical reading	**2.81**	**1.01**	**1.39 to 5.69**	**2.88**	**0.004**
Relevance [relevant] × comprehension	0.95	0.26	0.56 to 1.61	−0.18	0.855
Relevance [relevant] × technical reading	**1.99**	**0.54**	**1.17 to 3.40**	**2.53**	**0.011**
Random effects					
*σ* ^2^	3.29				
*τ*_00sentence_	0.19				
*τ*_00participant_	1.90				
*τ*_11sentence.comprehension.z_	0.04				
*ρ*_01sentence_	0.37				
ICC	0.39				
*N*_participant_	23				
*N*_sentence_	47				
Observations	518				
Marginal *R* ^2^/Conditional *R* ^2^	0.158/0.489				

*Note:* Technical reading and comprehension have been *z*‐transformed. Bolding denotes coefficients with *p* < 0.05.

**TABLE A9:** The final lm model for the number of relevant words.

Predictor	Estimate	SE	95% CI	*t*	*p*
Intercept	**5.41**	**0.28**	**4.81 to 6.01**	**19.14**	**< 0.001**
Technical reading	−0.53	0.39	−1.36 to 0.30	−1.35	0.196
Comprehension	0.65	0.33	−0.04 to 1.34	1.98	0.065
FD (int)	−0.22	0.29	−0.84 to 0.39	−0.77	0.453
FD (rel)	1.70	1.01	−0.44 to 3.85	1.68	0.112
*p*LB (int)	0.28	0.28	−0.30 to 0.87	1.03	0.319
*p*LB (rel)	−0.00	0.03	−0.07 to 0.06	−0.07	0.948
Observations	23				
*R* ^2^/*R* ^2^ adjusted	0.331/0.080				

*Note:* Technical reading, comprehension, and intercept variables have been *z*‐transformed. Bolding denotes coefficients with *p* < 0.05.

Abbreviations: FD, first‐pass duration; int., intercept; pLB, probability of a look‐back; rel, relevance.

**TABLE A10:** The final lm model for the coherence.

Predictor	Estimate	SE	95% CI	*t*	*p*
Intercept	**2.51**	**0.31**	**1.85 to 3.18**	**8.01**	**< 0.001**
Technical reading	−0.04	0.43	−0.96 to 0.88	−0.09	0.926
Comprehension	0.57	0.36	−0.19 to 1.34	1.59	0.132
FD (int)	−0.08	0.32	−0.77 to 0.60	−0.26	0.798
FD (rel)	−0.76	1.12	−3.14 to 1.63	−0.67	0.511
*p*LB (int)	−0.09	0.31	−0.74 to 0.56	−0.31	0.764
*p*LB (rel)	−0.01	0.03	−0.08 to 0.06	−0.32	0.756
Observations	23				
*R* ^2^/*R* ^2^ adjusted	0.214/−0.080				

*Note:* Technical reading, comprehension, and intercept variables have been *z*‐transformed. Bolding denotes coefficients with *p* < 0.05.

Abbreviations: FD, first‐pass duration; int., intercept; pLB, probability of a look‐back; rel, relevance.

**TABLE A11:** The final lm model for the conceptual science content.

Predictor	Estimate	SE	95% CI	*t*	*p*
Intercept	**3.88**	**0.44**	**2.93 to 4.82**	**8.72**	**< 0.001**
Technical reading	−0.23	0.62	−1.53 to 1.08	−0.37	0.719
Comprehension	0.32	0.51	−0.77 to 1.41	0.62	0.542
FD (int)	−0.13	0.46	−1.10 to 0.85	−0.27	0.787
FD (rel)	0.46	1.59	−2.92 to 3.84	0.29	0.775
*p*LB (int)	0.15	0.43	−0.77 to 1.07	0.34	0.739
*p*LB (rel)	0.03	0.05	−0.07 to 0.13	0.62	0.547
Observations	23				
*R* ^2^/*R* ^2^ adjusted	0.049/−0.307				

*Note:* Technical reading, comprehension, and intercept variables have been *z*‐transformed. Bolding denotes coefficients with *p* < 0.05.

Abbreviations: FD, first‐pass duration; int., intercept; pLB, probability of a look‐back; rel, relevance.
